# ERGA-BGE reference genome of *Xylophaga dorsalis - *a common deep-sea wood-boring bivalve with Atlantic-Mediterranean distribution

**DOI:** 10.12688/openreseurope.22692.2

**Published:** 2026-05-11

**Authors:** Sergi Taboada, Carlota Gracia-Sancha, Carles Galià-Camps, Astrid Böhne, Rita Monteiro, Thomas Marcussen, Rebekah A. Oomen, Torsten H. Struck, Marta Gut, Laura Aguilera, Francisco Câmara Ferreira, Fernando Cruz, Jèssica Gómez-Garrido, Tyler S. Alioto, Fergal Martin, Anna Lazar, Leanne Haggerty, Chiara Bortoluzzi

**Affiliations:** 1 Departamento de Biodiversidad y Biología Evolutiva, CSIC, Museo Nacional de Ciencias Naturales (MNCN), Madrid, 28006, Spain; 2 Museum Koenig Bonn, Adenauerallee 127, Leibniz Institute for the Analysis of Biodiversity Change, Bonn, 53113, Germany; 3 Natural History Museum, P.O. Box 1172, Blindern, University of Oslo, Oslo, 0318, Norway; 4 Centre for Ecological & Evolutionary Synthesis, Blindernveien 31, University of Oslo, Oslo, 0371, Norway; 5 Department of Biological Sciences, 100 Tucker Park Road, University of New Brunswick Saint John, Saint John, E2K5E2, Canada; 6 Tjärnö Marine Laboratory, Hättebäcksvägen 7, University of Gothenburg, Gothenburg, 45296, Sweden; 7 Centre for Coastal Research, Universitetsveien 25, University of Agder, Kristiansand, 4630, Norway; 8 Centro Nacional de Análisis Genómico (CNAG), Baldiri Reixac 4, Barcelona, 08028, Spain; 9 Universitat de Barcelona (UB), Barcelona, 08028, Spain; 10 European Molecular Biology Laboratory, European Bioinformatics Institute, Wellcome Genome Campus, Hinxton, CB10 1SD, UK; 11 SIB Swiss Institute of Bioinformatics, Amphipôle, Quartier UNIL-Sorge, Lausanne, 1015, Switzerland

**Keywords:** Xylophaga dorsalis, genome assembly, European Reference Genome Atlas, Biodiversity Genomic Europe, Earth Biogenome Project, Mollusca, Bivalvia, Xylophagaidae

## Abstract

*Xylophaga dorsalis* is a common Atlantic-Mediterranean mollusc that plays a crucial role in deep-sea habitats, where it digests wood that reaches the seabed through a unique symbiosis with specialised bacteria. The reference genome of *X. dorsalis* thus offers a crucial resource for uncovering the genetic basis of the species adaptability to wood bore in deep-water ecosystems. The entirety of the genome sequence was assembled into 18 contiguous chromosomal pseudomolecules (superscaffolds) and 1 mitochondrial genome. This chromosome-level assembly encompasses 0.451 Gb, composed of 1,259 contigs and 320 scaffolds, with contig and scaffold N50 values of 1.30 Mb and 25.4 Mb, respectively. The genome assembly encodes 19,441 protein-coding genes (34,405 transcripts) and 6,716 non-coding genes.

## Introduction

*Xylophaga dorsalis*, a mollusc from the family *Xylophagaidae*, is a deep-sea wood-boring bivalve commonly found in association with wood remains at depths ranging from 130 to 2,000 m depth ( Romano *et al*., 2014). *Xylophaga dorsalis* plays a crucial role in the deep sea, boring into and digesting wood that reaches the seabed, thus accelerating its decomposition. This activity releases recalcitrant organic matter retained in the wood, putting back nutrients into the benthic ecosystems, supporting diverse communities of opportunistic microbes and scavengers. Importantly, the burrowing of these bivalves creates complex ephemeral microhabitats that increase local biodiversity ( Bienhold *et al*., 2013). In this respect, *Xylophaga* species are functionally analogous to shallow-water wood-boring bivalves such as teredinids, although they operate in deep-sea environments where wood falls represent a major, yet patchy, source of organic input. While *X. dorsalis* is distributed across the Mediterranean and in the North-East Atlantic from the Cantabrian Sea to the northern part of Norway, previous reports of its occurrence in the North-West Atlantic are now in doubt ( Romano *et al*., 2014).

During evolution *Xylophaga* species have established a unique relationship with endosymbiotic bacteria found in the gills, which convert otherwise unavailable energy sources such as cellulose into forms readily metabolized by the host ( Distel & Roberts, 1997). Developing a high-quality reference genome for *X. dorsalis* is thus essential for advancing our understanding of its unique genetic makeup and adaptive traits and will shed light onto host-symbiont coevolutionary processes. A reference genome will thus provide the means to identify the genetic basis of metabolic complementarity between host and symbiont, offering insights into how these partnerships have evolved and are maintained.

The generation of this reference resource, which is the first and only one available for the *Xylophaga* genus as retrieved from Genomes on a Tree ( Challis *et al*., 2023), was coordinated by the European Reference Genome Atlas (ERGA) initiative’s Biodiversity Genomics Europe (BGE) project, supporting ERGA’s aims of promoting transnational cooperation to promote advances in the application of genomics technologies to protect and restore biodiversity ( Mazzoni *et al*., 2023).

## Materials & Methods

ERGA’s sequencing strategy includes Oxford Nanopore Technology (ONT) and/or Pacific Biosciences (PacBio) for long-read sequencing, along with Hi-C sequencing for chromosomal architecture, Illumina Paired-End (PE) for polishing (i.e. recommended for ONT-only assemblies), and RNA sequencing for transcriptomic profiling, to facilitate genome assembly and annotation.

### Sample and sampling information

On 28th of May 2023, Sergi Taboada and Carlota Gracia-Sancha sampled 50 specimens of *Xylophaga dorsalis* (sex unknown). Specimens were collected from a sunken tree log at a depth of 1,231 m near the shore of Mazarrón, Murcia, Spain, during the *LanderFleet Mazarrón 2023* sampling campaign aboard the Ramón Margalef vessel. Specimens were identified by Sergi Taboada based on barcoding a fragment of the cytochrome *c* oxidase subunit I gene and morphology following Romano *et al*. (2014). The biological material collected in Spain, and used to generate digital sequences, was retrieved from wildlife taxa regulated by the Spanish Royal Decree 124/2017. No ABS permits were granted as utilization of the material falls under the exemption in Article 3(2) of the Spanish Royal Decree 124/2017 and was confirmed by the national authorities. Specimens were caught with forceps and were euthanized by snap-frozing them directly in liquid nitrogen. Specimens were kept in liquid nitrogen until DNA extraction.

### Vouchering information

Physical reference materials for the sequenced specimen have been deposited in the Museo Nacional de Ciencias Naturales (MNCN-CSIC) https://www.mncn.csic.es/es under the accession number 15.07/20008.

Reference tissue material of the body wall is available from a proxy individual at the Biobank of the Museo Nacional de Ciencias Naturales (MNCN-CSIC) https://www.mncn.csic.es/es under the voucher ID MNCN-ADN 151.772.

### Genetic information

According to data retrieved from Genomes on a Tree ( Challis *et al*., 2023), the genome was expected to be diploid, with an expected haploid number of 19 chromosomes (2n = 38) and an estimated genome size based on the ancestral taxa *Cyrtopleura costata* of 0.68 Gb. This estimated genome size was used prior to sequencing to help calculate sequencing effort. After sequencing, counting of k-mers in the Illumina read data with Meryl (k = 20) ( Rhie *et al*., 2020) and genome size estimation with Genomescope 2.0 ( Ranallo-Benavidez *et al*., 2020) gives an estimated haploid genome size of 454 Mb.

### DNA/RNA processing

DNA was extracted from one whole specimen using the Blood & Cell Culture DNA Mini Kit (Qiagen) following the manufacturer’s instructions. DNA quantification was performed using a Qubit dsDNA BR Assay Kit (Thermo Fisher Scientific), and DNA integrity was assessed using a Genomic DNA 165 Kb Kit (Agilent) on the Femto Pulse system (Agilent). The DNA was stored at +4 °C until sequenced.

RNA was extracted from the whole organism using a RNeasy Mini Kit (Qiagen) according to the manufacturer’s instructions. RNA quantification was performed using the Qubit RNA BR kit, and RNA integrity was assessed using a Bioanalyzer 2100 system (Agilent) RNA 6000 Nano Kit (Agilent). The RNA was stored at −80 °C until sequenced.

### Library preparation and sequencing

For long-read whole genome sequencing, a library was prepared using the SQK-LSK114 Kit (Oxford Nanopore Technologies, ONT), which was then sequenced in one R10.4.1 flow cell on a PromethION 24 A Series instrument (ONT). A short-read whole-genome sequencing library was prepared using the KAPA Hyper Prep Kit (Roche). A Hi-C library was prepared from the whole specimen using the Dovetail Omni-C Kit (Cantata Bio), followed by the KAPA Hyper Prep Kit for Illumina sequencing (Roche). The RNA library was prepared using the KAPA mRNA Hyper prep kit (Roche). All short-read libraries were sequenced on a NovaSeq 6000 instrument (2x150bp, Illumina). In total 93x Oxford Nanopore, 230x Illumina WGS shotgun, and 178x HiC data were sequenced to generate the assembly.

### Genome assembly methods

The genome was assembled using the CLAWS pipeline ( Gomez-Garrido, 2024). Briefly, reads were pre-processed for quality and length using Trim Galore v0.6.7 ( http://www.bioinformatics.babraham.ac.uk/projects/trim_galore/) and Filtlong v0.2.1 ( https://github.com/rrwick/Filtlong), and initial contigs were assembled using Flye v2.9.1-b1780 ( Kolmogorov *et al*., 2019) with default parameters, followed by polishing of the assembled contigs using HyPo v1.0.3 ( Kundu *et al*., 2019), and removal of retained haplotigs using purge-dups v1.2.6 ( Guan *et al*., 2020). Six short contigs (<100 kb) corresponding to bacterial sequences (Pseudomonadota, Bacillota) were identified with BlobToolKit v0.6.0 ( Challis *et al*., 2020) and removed from the assembly. The scaffolded assembly, which spans a total length of 451,816,200 bp, was obtained by scaffolding the contig-level assembly with Hi-C data using YAHS v1.2a ( Zhou *et al*., 2023) followed by manual curation in PretextView v0.2.5 ( https://gitlab.com/wtsi-grit/rapid-curation ) to remove any false joins and incorporate any sequences not automatically scaffolded into their respective locations in the chromosomal pseudomolecules (or super-scaffolds). Finally, the mitochondrial genome was assembled using ONT reads as a single circular contig of 21,574 bp using the FOAM pipeline ( https://github.com/cnag-aat/FOAM) and included in the released assembly (GCA_965225065.2). BUSCO v5.8.0 was used to estimate the level of genome completeness on the final assembly using the Eukaryota database (odb10), whereas Merqury v1.3 ( Rhie *et al*., 2020) was used to estimate base accuracy Quality Value. Summary analysis of the released assembly was performed using the ERGA-BGE Genome Report ASM Galaxy workflow ( https://doi.org/10.48546/workflowhub.workflow.1103.2).

### Genome annotation methods

A gene set was generated for a previously released chromosome-level genome assembly (GCA_965225065.1) using the Ensembl Gene Annotation system ( Aken *et al*., 2016), primarily by aligning publicly available short-read RNA-seq data from BioSample SAMEA116055609 to the genome. Gaps in the annotation were filled via protein-to-genome alignments of a select set of clade-specific proteins from ( UniProt Consortium, 2019) which had experimental evidence at the protein or transcript level. At each locus, data were aggregated and consolidated, prioritising models derived from RNA-seq data, resulting in a final set of gene models and associated non-redundant transcript sets. To distinguish true isoforms from fragments, the likelihood of each open reading frame (ORF) was evaluated against known metazoan proteins. Low-quality transcript models, such as those showing evidence of fragmented ORFs, were removed. In cases where RNA-seq data were fragmented or absent, homology data were prioritised, favouring longer transcripts with strong intron support from short-read data. The resulting gene models were classified into two categories: protein-coding, and long non-coding. Models that did not overlap protein-coding genes and were constructed from transcriptomic data were considered potential lncRNAs. Potential lncRNAs were further filtered to remove single-exon loci due to their unreliability. Putative miRNAs were predicted by performing a BLAST search of miRBase ( Kozomara *et al*., 2019) against the genome, followed by RNAfold analysis ( Gruber *et al*., 2008). Other small non-coding loci were identified by scanning the genome with Rfam ( Kalvari *et al*., 2018) and passing the results through Infernal ( Nawrocki & Eddy, 2013). Summary analysis of the released annotation was carried out using the ERGA-BGE Genome Report ANNOT Galaxy workflow ( 10.48546/workflowhub.workflow.1096.1).

## Results

### Genome assembly

**Figure 1.**
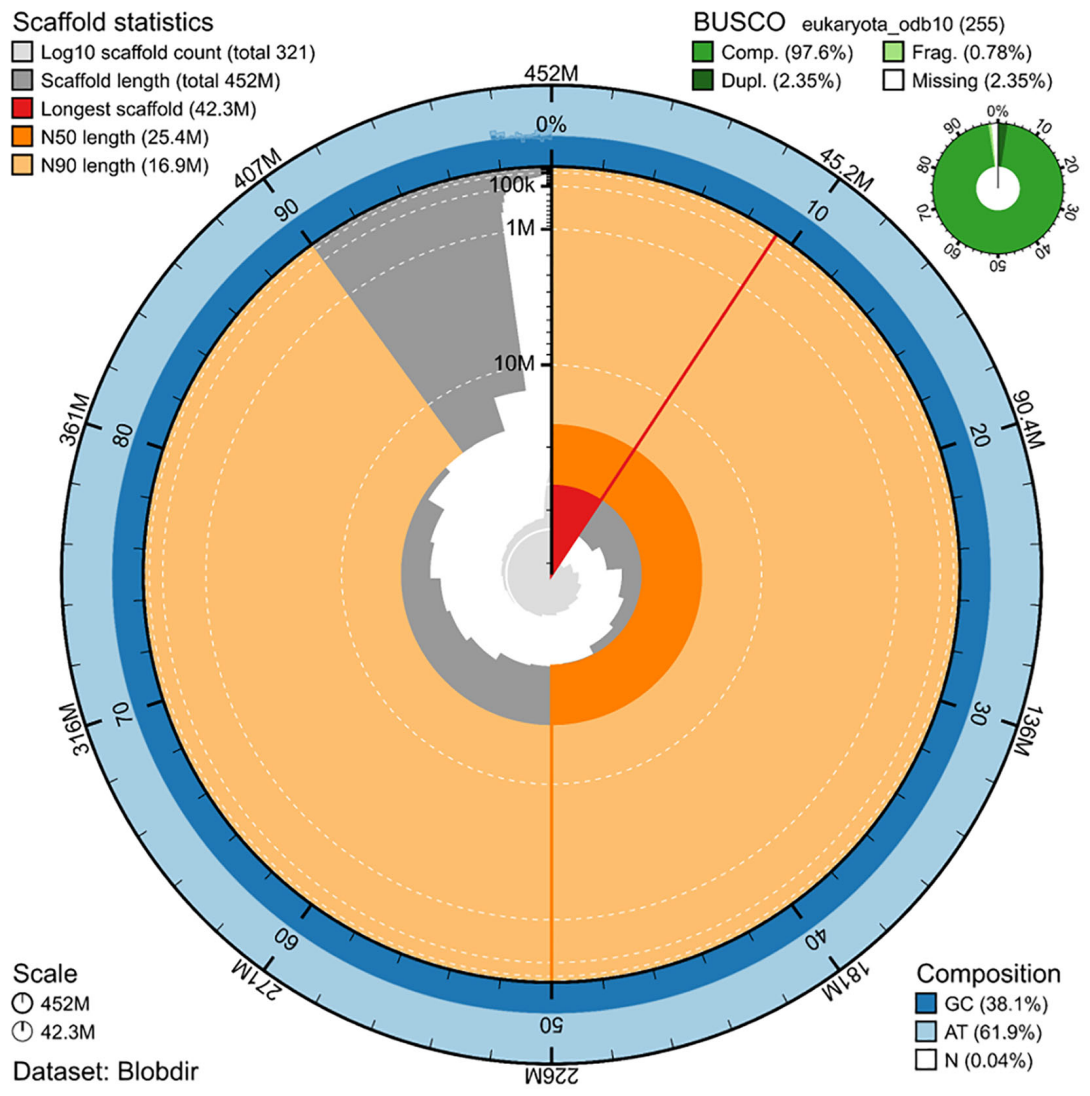
Snail plot summary of assembly statistics. The main plot is divided into 1,000 size-ordered bins around the circumference, with each bin representing 0.1% of the 451,816,200 bp assembly including the mitochondrial genome. The distribution of sequence lengths is shown in dark grey, with the plot radius scaled to the longest sequence present in the assembly (42.3 Mb, shown in red). Orange and pale-orange arcs show the scaffold N50 and N90 sequence lengths (25,438,142 and 16,851,157 bp), respectively. The pale grey spiral shows the cumulative sequence count on a log-scale, with white scale lines showing successive orders of magnitude. The blue and pale-blue area around the outside of the plot shows the distribution of GC, AT, and N percentages in the same bins as the inner plot. A summary of complete, fragmented, duplicated, and missing BUSCO genes found in the assembled genome from the Eukaryota database (odb10) is shown in the top right.

**Figure 2.**
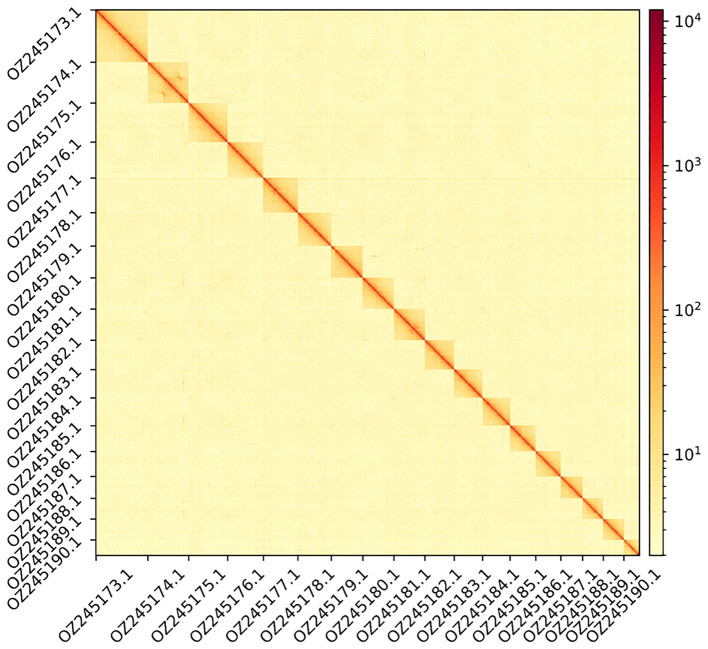
Hi-C contact map showing spatial interactions between regions of the genome. The diagonal corresponds to intra-chromosomal contacts, depicting chromosome boundaries. The frequency of contacts is shown on a logarithmic heatmap scale. Hi-C matrix bins were merged into a 300 kb bin size for plotting. For visualisation purposes, the Hi-C contact map shows only the 18 chromosomes.

The genome was assembled with Flye, polished, purged and scaffolded and curated as detailed in the methods. The curated assembly comprises 18 superscaffolds and 286 scaffolds that could not be placed into any superscaffold, and the mitogenome ( Figures 1 & 2). The assembly has a GC content of 38.1%, a contig N50 of 1,253,263 bp (L50 = 85), and a scaffold N50 of 25,438,142 bp (L50 = 8). A total of 939 gaps are present, with a cumulative gap size of 187.8 kb. Completeness assessed using BUSCO ( Manni *et al*., 2021) against the Eukaryota database (odb10) was 97.6% (95.3% single-copy and 2.4% duplicated). Finally, the assembly has a base accuracy Quality Value (QV) or 49.7 and a kmer completeness of 68% as calculated by Merqury ( Rhie *et al*., 2020).

### Genome annotation

The genome annotation consists of 19,441 protein-coding genes with associated 34,405 transcripts, in addition to 6,716 non-coding genes ( Table 1). BUSCO was run using the Mollusca database (odb12) on the canonical proteins to avoid high duplication caused by isoforms. This led to a completeness score of 95.9%. Using the OMAmer Metazoa-v2.0.0.h5 database for OMArk ( Nevers *et al*., 2025) resulted in 94.1% completeness and 58.2% consistency ( Table 2).

**Table 1.** Statistics from assembled gene models.

	No. genes	No. transcripts	Mean gene length (bp)	No. single-exon genes	Mean exons per transcript
**mRNA**	19,441	34,405	15,708	568	9.9
**pseudogene**	0.0	0.0	0.0	0.0	0.0
**snoRNA**	162	162	112	162	1.0
**lncRNA**	5,237	6,113	5,253	54	2.3
**tRNA**	1,046	1,046	75	1,046	1.0
**snRNA**	128	128	152	128	1.0
**rRNA**	141	141	407	141	1.0
**scRNA**	2	2	132	2	1.0

**Table 2.** Annotation completeness and consistency scores calculated by BUSCO run in protein mode (mollusca_odb12) and OMArk (Metazoa-v2.0.0.h5).

	Complete	Singular	Duplicated	Fragmented	Missing
**BUSCO**	4,241 (95.9%)	4,173 (94.4%)	68 (1.5%)	116 (2.6%)	64 (1.4%)
**OMArk**	2,027 (94.1%)	364 (16.9%)	1,663 (77.2%)	-	126 (5.8%)

	Consistent	Inconsistent	Contaminants	Unknown	
**OMArk**	23,118 (58.2%)	4,160 (10.5%)	0 (0.0%)	12,442 (31.3%)	

## Data availability

*Xylophaga dorsalis* and the related genomic study were assigned to Tree of Life ID (ToLID) ‘xbXylDors1’ and all sample, sequence, and assembly information are available under the umbrella BioProject PRJEB87517. The sample information is available at the following BioSample accessions: SAMEA116055604, SAMEA116055609, and SAMEA116055611. The genome assembly is accessible from ENA under accession number GCA_965225065.2. This version includes the mitogenome, while the first version GCA_965225065.1 includes only the nuclear genome. The gene annotation is on the Ensembl webpage ( https://projects.ensembl.org/erga-bge/). Sequencing data produced as part of this project are available from ENA at the following accessions: ERX14171855, ERX14171856, ERX14171857, and ERX14171858. Documentation related to the genome assembly and curation can be found in the ERGA Assembly Report (EAR) document available at https://github.com/ERGA-consortium/EARs/blob/main/Assembly_Reports/Xylophaga_dorsalis/xbXylDors1/. Further details and data about the project are hosted on the ERGA portal at https://portal.erga-biodiversity.eu/data_portal/1526741.

## Author contributions

ST coordinated the project; ST, CG-S and CG-C collected the species; ST, CG-S and CG-C identified the species; ST, CG-S and CG-C sampled and preserved biological material and provided metadata; AsB, RM, TM, RAO, and THS provided support in sampling, shipping of biological material, metadata collection, and management; LA and MG extracted DNA, prepared libraries, and performed sequencing; FCF, FC, JG-G, and TSA performed genome assembly and curation under the supervision of TSA; FM, AL, and LH performed genome annotation; CB generated the analysis and report. All authors contributed to the writing, review, and editing of this genome note and read and approved the final version.
