# Supplementary material for: ERGA-BGE reference genome of Xylophaga dorsalis - a common deep-sea wood-boring bivalve with Atlantic-Mediterranean distribution
Source: Open Res Eur. 2026 May 11;6:33. Originally published 2026 Jan 30. [Version 2] doi: 10.12688/openreseurope.22692.2 (PMC13173280; doi:10.12688/openreseurope.22692.2)
Supplement: Peer review history

# Reviewer report: Reviewer response for version 2

**DOI:** 10.21956/openreseurope.25825.r74036

**Published:** 2026-05-13

**Authors:** Fabrizio Ghiselli

**Affiliations:** 1 University of Bologna, Bologna, Italy

The Authors have addressed the concerns I raised in my peer review report appropriately.

 I have no further comments to make.

Are sufficient details of methods and materials provided to allow replication by others?

Yes

Is the rationale for creating the dataset(s) clearly described?

Yes

Are the datasets clearly presented in a useable and accessible format?

Partly

Are the protocols appropriate and is the work technically sound?

Yes

Reviewer Expertise:

Comparative Genomics, Genome Evolution, Molecular Evolution, Mitochondria, Biodiversity Genomics

I confirm that I have read this submission and believe that I have an appropriate level of expertise to confirm that it is of an acceptable scientific standard.

# Reviewer report: Reviewer response for version 1

**DOI:** 10.21956/openreseurope.24540.r70658

**Published:** 2026-03-19

**Authors:** Fabrizio Ghiselli

**Affiliations:** 1 University of Bologna, Bologna, Italy

This manuscript presents a chromosome-level genome assembly of *Xylophaga dorsalis*, a species of ecological importance due to its role in wood degradation in deep-sea ecosystems. The dataset represents a valuable resource for the research community. Several aspects of the manuscript would benefit from clarification and additional details. Addressing these points would substantially strengthen the work and improve its clarity and reproducibility prior to publication.

 I share several of the concerns outlined by Reviewers 1 and 3. My comments are provided below.

 - Genome Size Estimation and Assembly Validation

 The genome size of *X. dorsalis* is estimated based on a closely related species. However, given the increasing evidence of high variability in genome size even among closely related species (and even within species), extrapolation from related taxa is not recommended. Genome size and ploidy should be estimated directly from the sequencing data using k-mer–based approaches (e.g., GenomeScope or similar).

 In addition, it would be useful to provide a table including the following:

 A k-mer profile.

 Coverage statistics.

 Heterozygosity estimates.

 Assembly quality metrics (e.g., N50, L50, total assembly size, number of contigs/scaffolds).

 BUSCO results, standardized using a single database version and clearly linked to the assembly version analyzed.

 If Merqury was used, full statistics should be described in the Methods.

 Most of these metrics appear to be shown in the snail plot; however, a summary table would be easier to read and interpret.

 - Inconsistencies in Annotation Statistics: as noted by Reviewers 1 and 3, I also observed inconsistencies between the annotation statistics reported in the Abstract and those presented in the Results and Table 1. These discrepancies should be carefully reviewed and corrected to ensure consistency throughout the manuscript.

 - Transcriptome Results Should Be Described in Greater Detail. Additional information would greatly improve clarity and reproducibility.

 For example:

 The number of RNA-seq samples used.

 Assembly statistics.

 Whether genome-guided and/or de novo transcriptome assemblies were performed.

 How RNA-seq data were integrated into gene model construction.

 Biosample IDs (e.g., SAMEA116055609), which could not be accessed or verified.

 Total reads per sample.

 Assembly size and N50 (if de novo assembled).

 BUSCO completeness of the transcriptome.

 Mapping rates to the genome.

 Providing these details would strengthen the annotation section considerably.

 - Mitochondrial Genome

 Regarding Reviewer #1’s concern about the mitochondrial genome size appearing larger than expected compared to published *X. dorsalis* mitochondrial data (~16.8 kb), I would like to add a comment.

 I have worked on bivalve mitochondrial genomics for 20 years, and mitochondrial genome size can be quite variable, even within species, often due to repeats localized upstream or downstream of the control region. It is also important to consider that this study used long-read sequencing. If the previously published genome was generated using short-read data, repeat regions may have been collapsed, resulting in an underestimated genome size. Therefore, the observed size difference may reflect biological reality rather than an assembly artifact.

 That said, given the peculiarities of mitochondrial biology in bivalves, Reviewer #1’s suggestions for careful verification are appropriate. There is also an additional related issue to consider. Many (certainly over 100) bivalve species exhibit Doubly Uniparental Inheritance (DUI) of mitochondria. DUI species possess two distinct mitochondrial lineages with separate inheritance patterns (maternal and paternal). These lineages can be highly divergent (up to 0.45 nucleotide p-distance), raising the possibility of chimeric assemblies or other assembly complications. DUI can be difficult to detect. In some cases, it is suggested by unusually high heterozygosity in mitochondrial sequences; in others, two clearly distinct mitochondrial genomes may assemble separately.

 While assessing DUI is beyond the scope of this genome report, if the authors identify any signals or indications suggesting its presence, reporting this would be valuable for the community.

 I also found that the links to the MNCN website do not function, and voucher specimens could not be located using the search tool.

Are sufficient details of methods and materials provided to allow replication by others?

Yes

Is the rationale for creating the dataset(s) clearly described?

Yes

Are the datasets clearly presented in a useable and accessible format?

Partly

Are the protocols appropriate and is the work technically sound?

Yes

Reviewer Expertise:

Comparative Genomics, Genome Evolution, Molecular Evolution, Mitochondria, Biodiversity Genomics

I confirm that I have read this submission and believe that I have an appropriate level of expertise to confirm that it is of an acceptable scientific standard, however I have significant reservations, as outlined above.

# Response

**Published:** 2026-04-30

**Authors:** Chiara Bortoluzzi

**Affiliations:** Swiss Institute of Bioinformatics, Lausanne, Vaud, Switzerland

We thank the reviewer for their comments. We have addressed all of them as below:

1. We understand the confusion. The genome size estimated using related species was done only to estimate the amount of sequencing required prior to sequencing. We rephrase this in the main text. 
2. All of these statistics are already reported in the ERGA Assembly Report (EAR, https://github.com/ERGA-consortium/EARs/blob/main/Assembly_Reports/Xylophaga_dorsalis/xbXylDors1/) cited in the manuscript. Hence, we did not see the point of reporting this information once again as the reader is referred to the EAR in the main text. 
3. We now report Merqury in our Materials and methods section
4. We have now corrected the genome annotation results throughout the manuscript
5. The text reported in the main text for the genome annotation section follows the standard Ensembl template. It follows that most Ensembl results are reported in their website. In the case of *X. dorsalis*, the full annotation statistics can be found here: https://beta.ensembl.org/species/84d8aa9e-1f9e-40fe-a304-b3d2d5df1e9a. Regarding BioSample SAMEA116055609, this can be accessed here: https://www.ebi.ac.uk/biosamples/samples/SAMEA116055609. 
6. The links to the MNCN are meant to redirect the reader to the National History Museum and not to the voucher itself. To access the voucher a request should be submitted to the Museum. 

# Reviewer report: Reviewer response for version 1

**DOI:** 10.21956/openreseurope.24540.r70657

**Published:** 2026-03-12

**Authors:** Melody S Clark

**Affiliations:** 1 British Antarctic Survey, Cambridge, UK

The ecological role of Xylophaga dorsalis is well described and the genome has been subjected to comprehensive sequencing. However, I have a number of concerns.

 Is this an updated genome assembly based on GCA_965225065.1? This is unclear, whether this is an updated reference genome based on these previous data or a completely separate one?

 Firstly none of the links to the mncn website work in terms of access to the information on the voucher specimens. I could not find the specimens using the mncn search function. There needs to be pictures of the voucher specimens included in this publication and links to the specimens in mncn need to be direct links.

 The genome size of this species is estimated using related species, but this must be based on actual data (k-mer-based analysis as per Reviewer 1), as I am sure the authors are aware of the c-value paradox.

 The data from each individual sequencing method must be presented in a table.

 The workflows are not clear. Links to centralised generic workflows with multiple pathways are very confusing and the authors need to explicitly state how they generated both the genome assembly and the  annotation, so that these can be replicated.

 More data are needed on the RNA-seq: assembly stats etc, especially as I could not access the Biosample data typing in the IDs provided in this publication e.g SAMEA116055609. Furthermore, I am not convinced about the use of clade-specific proteins for annotation. The authors should generate more RNA-seq data if required for annotation.

 The data in Table 1 do not correlate with the abstract and needs correction.

 The authors state that they generated 320 scaffolds, but these ultimately resulted in 18 chromosomal psuedochromosomes, plus the mitochondrial genome. The authors need to state the the contig and scaffold data for each pseudochromosome.

Are sufficient details of methods and materials provided to allow replication by others?

No

Is the rationale for creating the dataset(s) clearly described?

Yes

Are the datasets clearly presented in a useable and accessible format?

Partly

Are the protocols appropriate and is the work technically sound?

Yes

Reviewer Expertise:

Transcriptomics and genomics of non-model species

I confirm that I have read this submission and believe that I have an appropriate level of expertise to confirm that it is of an acceptable scientific standard, however I have significant reservations, as outlined above.

# Response

**Published:** 2026-04-30

**Authors:** Chiara Bortoluzzi

**Affiliations:** Swiss Institute of Bioinformatics, Lausanne, Vaud, Switzerland

We thank the reviewer for their comments. We have addressed all of them as below:

1. The genome assembly for which we report on is an improved version of GCA_965225065.1. We specified this in the main text.
2. The links to the MNCN are meant to redirect the reader to the National History Museum and not to the voucher itself. To access the voucher a request should be submitted to the Museum.
3. We understand the confusion on the estimated genome size. The genome size estimated via GoaT was used to estimate the amount of sequencing required prior to sequencing. 
4. As suggested, we expanded the genome assembly section to ensure replicability. With regard to the genome annotation part, the text reported in the main text follows the standard Ensembl template.
5. All BioSamples data can be access either via ENA ( https://www.ebi.ac.uk/ena/browser/view/SAMEA116055609) or BioSamples ( https://www.ebi.ac.uk/biosamples/samples/SAMEA116055609). We cannot generate additional RNA-Seq data because the BGE project has ended as well as funding. 
6. We indeed realised the mistake and have now corrected the Abstract to meet the results presented in Table 1. 
7. Following Hi-C mapping to a flye assembly and manual curation in Pretext View, the final scaffolded assembly submitted to the ENA comprises 18 SUPER scaffolds (pseudochromosomes) and 286 smaller scaffolds that could not be placed onto any pseudochromosome, for a total of 320 sequences. We specified this in the main text.

# Reviewer report: Reviewer response for version 1

**DOI:** 10.21956/openreseurope.24540.r70656

**Published:** 2026-03-05

**Authors:** Fabrizio Marcondes Machado

**Affiliations:** 1 Universidade Estadual de Campinas, Campinas, Brazil

The study represents a valuable genomic resource for marine biology and deep-sea ecology. The chromosome-level assembly is technically robust, with consistent N50 statistics, and the gene annotation provides a high-density dataset for future functional and comparative studies. The work addresses an ecologically significant species and offers insights into the genetic basis of its symbiosis and wood-digestion capabilities.

 Discussion: However, some aspects require clarification or further elaboration. Some numbers, while plausible, could benefit from additional context or comparison with related bivalves. Although this is a study with a very specific focus, it is not clear whether a complete genome of another species in the same genus or family already exists. Furthermore, while the manuscript mentions the ecological role of *X. dorsalis*, it could benefit from a comparative perspective with other deep-sea bivalves or wood-boring species, perhaps even regarding the number of genes identified.

 Introduction: Although I understand how a high-quality genome can help us understand host-symbiont coevolutionary processes, I would suggest that the authors include a brief explanatory sentence here. I am confident that doing so would benefit readers, especially younger researchers, by clarifying why this genome is particularly valuable for studying these interactions.

Are sufficient details of methods and materials provided to allow replication by others?

Yes

Is the rationale for creating the dataset(s) clearly described?

Yes

Are the datasets clearly presented in a useable and accessible format?

Yes

Are the protocols appropriate and is the work technically sound?

Yes

Reviewer Expertise:

Morphology and anatomy of bivalves, Taxonomy of marine molluscs, Genomics of bivalves.

I confirm that I have read this submission and believe that I have an appropriate level of expertise to confirm that it is of an acceptable scientific standard, however I have significant reservations, as outlined above.

# Response

**Published:** 2026-04-30

**Authors:** Chiara Bortoluzzi

**Affiliations:** Swiss Institute of Bioinformatics, Lausanne, Vaud, Switzerland

We thank the reviewer for their comments. We have addressed all of them as below:

1. We now specify in the introduction that the reference genome of *X. dorsalis *is the first and only one available for the *Xylophaga *genus. 
2. We now included a brief explanatory sentence in the Introduction explaining how a high-quality genome can help us understand host-symbiont co-evolutionary processes.

# Reviewer report: Reviewer response for version 1

**DOI:** 10.21956/openreseurope.24540.r69058

**Published:** 2026-02-10

**Authors:** Abdelmalek Lekired

**Affiliations:** 1 University of New Mexico, Albuquerque, NM, USA

**Reviewer Report:**

 We thank the authors for this valuable contribution to molluscan genomics, particularly for marine and deep-sea molluscs, which remain underrepresented in genomic datasets. The authors present a chromosome-level assembly and annotation of *Xylophaga dorsalis* as a data note. Overall, the work is potentially valuable and merits publication; however, several concerns regarding the methodology and the consistency of the reported data must be addressed before acceptance.

 Major Comments

**Abstract**

- The authors report 18 chromosomes and one mitochondrial genome; however, key genomic metrics are missing, including genome size, sequencing coverage, heterozygosity, BUSCO completeness, and general assembly statistics.
- Transcriptome metrics are not provided, and the annotation numbers reported in the abstract are inconsistent with those presented in the Results section.
- It is unclear whether the annotation was performed on the chromosome-level assembly or on a scaffold-level assembly.
- The number of contigs is mentioned in the abstract but not clearly reported in the Results section.

**Recommendation:** All reported metrics should be based consistently on the chromosome-level assembly and clearly stated.

**Results – Genome Assembly**

- The estimated genome size based on a closely related species is not sufficiently reliable. Genome size and ploidy should be estimated directly using k-mer–based analyses.
- Assembly statistics (e.g., N50, total assembly size, coverage, BUSCO, Merqury if used) should be clearly reported in Material and methods.

**Genome Annotation**

- There is a major inconsistency between annotation statistics reported in the abstract and those in the Results:
  - **Abstract:** 39,720 protein-coding genes (70,084 transcripts) and 12,153 non-coding genes.
  - **Results:** 19,441 protein-coding genes with 34,405 transcripts and 6,716 non-coding genes.

These discrepancies must be resolved and explained.

- The annotation methodology is not described clearly enough to be reproducible. Critical details are missing, including:
  - Whether the genome was soft-masked prior to annotation.
  - Which genome assembly version (contig, scaffold, or chromosome level) was used.
  - Whether ab initio gene predictors (e.g., AUGUSTUS) or RNA-Seq–based annotation tools were used.
- RNA-Seq usage is unclear:
  - How many RNA-Seq samples were used?
  - Were transcript models generated using genome-guided and/or de novo transcriptome assemblies?
  - How were these data integrated into the annotation?
- The Methods section reports results that are not described methodologically, including BUSCO and Merqury analyses.
- Two different BUSCO database versions are mentioned (odb10 for Metazoa and odb12 for Mollusca) without justification. This needs clarification and standardization.

**Recommendation:** Provide a clear, step-by-step annotation pipeline, ideally supported by a flowchart, and list all software tools and versions used.

**Mitochondrial Genome**

- The reported mitochondrial genome size appears suspiciously large, exceeding the known *Xylophaga dorsalis* mitochondrial genome by ~5 kb (GenBank: OM910830.1; 16,787 bp).
- The mitochondrial assembly should be carefully checked for duplication or assembly artifacts.

**Contamination Assessment**

- The authors report only six short bacterial contigs identified using BlobToolKit, but it is unclear:
  - Which assembly version was used for this analysis (contigs, scaffolds, or chromosome-level assembly)?
  - How contaminants were identified and removed in practice.
- It should be clarified whether decontamination was performed before or after chromosome-level scaffolding.

**Missing or Unclear Information**

 The manuscript would benefit from the following clarifications and additions:

1. A chronological and clearly described genome assembly workflow.
2. A reproducible genome annotation pipeline, including a flowchart.
3. Summary annotation statistics per chromosome.
4. The draft genome size prior to BlobToolKit filtering and chromosome-level assembly.
5. The number/percentage of contigs/sequences not anchored to chromosomes.

**Overall Recommendation**

 The dataset has clear potential value for the community; however, substantial revisions are required to improve methodological transparency, data consistency, and reproducibility. Addressing the points above will significantly strengthen the manuscript and its utility as a genomic resource.

Are sufficient details of methods and materials provided to allow replication by others?

No

Is the rationale for creating the dataset(s) clearly described?

Partly

Are the datasets clearly presented in a useable and accessible format?

Partly

Are the protocols appropriate and is the work technically sound?

Yes

Reviewer Expertise:

My expertise is in genome assembly, curation, and functional annotation of bacterial, fungal, and molluscan genomes, with a particular focus on non-model organisms. I have extensive experience generating and evaluating hybrid genome assemblies using short-read and long-read sequencing data, and integrating RNA-seq evidence to support gene prediction and transcript validation. My work includes assembly quality assessment, resolution of complex genomic features such as repeats, heterozygosity, and ploidy, and evidence-based gene annotation using RNA-seq–guided pipelines. I routinely analyze RNA-seq data for genome annotation, expression profiling, and locus-level interpretation, with an emphasis on producing biologically robust genome resources suitable for comparative and functional genomics.

I confirm that I have read this submission and believe that I have an appropriate level of expertise to confirm that it is of an acceptable scientific standard, however I have significant reservations, as outlined above.

## References

[1] : Whole-genome sequencing and genome-wide transcriptome profiling of the freshwater planorbid snail Bulinus ugandae (Mollusca: Gastropoda), a Nilotic bulinine refractory to Schistosoma haematobium. *BMC Genomics* .2025;26(1) : 10.1186/s12864-025-12320-3 10.1186/s12864-025-12320-3 41361373 PMC12690972

[2] : Comparative genomics reveals the acquisition of mobile genetic elements by the plant growth-promoting Pantoea eucrina OB49 in polluted environments. *Genomics* .2023;115(2) : 10.1016/j.ygeno.2023.110579 10.1016/j.ygeno.2023.110579 36792019

[3] : Exploring the genome-wide transcriptomic responses of Bulinus truncatus to Schistosoma haematobium infection: An important host-parasite system involved in the transmission of human urogenital schistosomiasis. *Molecular Immunology* .2024;175: 10.1016/j.molimm.2024.09.006 74-88 10.1016/j.molimm.2024.09.006 39307031 PMC12019995

[4] : First insights into the microbiome of Tunisian Hyalomma ticks gained through next-generation sequencing with a special focus on H. scupense. *PLOS ONE* .2022;17(5) : 10.1371/journal.pone.0268172 10.1371/journal.pone.0268172 PMC911955935587930

[5] : Genomic basis of schistosome resistance in a molluscan vector of human schistosomiasis. *iScience* .2025;28(1) : 10.1016/j.isci.2024.111520 10.1016/j.isci.2024.111520 PMC1169975539758819
